# Investigation of the Effects of an Online Support Group for Mental Health Problems on Stigma and Help-Seeking Among Japanese Adults: Cross-sectional Study

**DOI:** 10.2196/21348

**Published:** 2021-09-07

**Authors:** Osamu Kobori, Naoki Yoshinaga

**Affiliations:** 1 Department of Psychology International University of Health and Welfare Tokyo Japan; 2 School of Nursing Faculty of Medicine University of Miyazaki Miyazaki Japan

**Keywords:** online support group, mental health, depression, stigma, help-seeking

## Abstract

**Background:**

Online support groups vary widely in both goals and structures owing to the rapid development of social networking services. Several studies have shown the potential effectiveness of online support groups, such as reducing psychological distress (eg, depression) among individuals with mental health problems. However, online support groups often do not aim at effectiveness regarding distress relief–related outcomes.

**Objective:**

This study aims to examine whether the use frequency of online support group platform functions (U2plus) is associated with lower stigma and higher consumer activation.

**Methods:**

A total of 350 U2plus users participated in a web-based survey. They were asked what therapy they had received in the past and how often they logged on to it, used each of its functions, and completed the following questionnaires: the Patient Health Questionnaire-9, the Devaluation-Discrimination Scale, and the General Help-Seeking Questionnaire.

**Results:**

Regarding the therapy received, 88% (308/350) of participants had taken medication for mental health problems, and 66.6% (233/350) had received psychotherapy or mental health counseling. Regarding use frequency, 21.7% (74/341) of the participants signed in to U2plus and used its functions more than once a week. The use frequency of U2plus functions was not correlated with perceived stigma, but the use frequency of some functions was weakly correlated with help-seeking intentions from formal sources (eg, doctors and psychologists). However, multiple regression analyses revealed that the use frequency of those functions did not uniquely predict help-seeking intentions.

**Conclusions:**

It was suggested that online support groups may serve as an alternative treatment option for those who are already undergoing pharmacological treatment and are willing to seek help from whatever source they deem helpful.

## Introduction

### Cognitive Behavioral Therapy and the Internet

Andersson [1] argues that it is essential to develop alternatives to face-to-face treatments for three reasons: (1) there are not enough experienced clinicians who are able to provide evidence-based treatment to all those who need it; (2) some people cannot access specialist clinics and general practitioners who provide and are proficient in cognitive behavioral therapy (CBT) owing to the distance from the services to where they live; and (3) some people prefer web-based over face-to-face treatments. The first reason may help reduce the stigma related to mental health problems. The development of internet-based CBT and the rapid growth of internet access worldwide may potentially tackle these barriers to receiving psychological treatment.

After conducting a comprehensive review, Barak et al [2] classified internet-supported interventions, which are not limited to CBT, into four categories based on their primary approaches: (1) web-based interventions, defined as “a primarily self-guided intervention program that is executed by means of a prescriptive online program operated through a website and used by consumers seeking health- and mental-health-related assistance.” Barak et al [2] also remarked that web-based interventions attempt to create positive change or enhance knowledge, awareness, and understanding by providing sound health-related material and the use of interactive web-based components; (2) web-based counseling and therapy; (3) internet-operated therapeutic software; and (4) other web-based activities. Specifically, our study attempts to closely investigate online support groups in the fourth category.

### Alternative Effects of Online Support Groups

People with mental illness are often interested in and willing to form connections with others through social media; specifically, young adults with mental illness were more likely to express their personal views through blogging, form friendships via social media, and connect with people via the web who share the same interests [3]. In the case of individuals with schizophrenia, despite having fewer offline relationships and less internet access, had similar tendencies to form web-based social connections with those of adults without mental illness [4]. Furthermore, online support groups, forums, and chat rooms were shown to help people to discuss their sensitive mental health conditions [5] and seek and share information related to symptoms and medications [6]. Thus, there is a bulk of the literature showing the predisposition highlighted in the first line of this section, one that could be vital during times when social distancing is required, for example, the COVID-19 pandemic has brought upon such requirements and currently persists worldwide.

After this initial phase of social networking development and group establishment (ie, people getting in touch with others and entering groups), online support groups can take on different forms. First, they have different functions; they can provide emotional, informational, and instrumental support or even (and often) a combination of all three. In particular, support groups (online) may decrease isolation, increase health information sharing, and provide role modeling [7]. Second, online support groups can have different means of access; they may be closed, open only by invitation, or even open for all. In addition, their member structure is often not fixed, so members may come and go, and many (often most) are not active; instead, they are often rather passive observers of the online discussions. Third, groups can be either self-guided by facilitators or have professionals who monitor the discussions.

Some studies have analyzed the benefits of online support groups in depth; one remarks that writing about one’s feelings and experiences associated with life challenges decreases one’s negative emotions, writing about neutral events has no such effect, and sharing one’s bad feelings with others has a tremendous relieving effect [8]. Thus, group participants may feel better when they can share their difficult emotions with others who are available to listen and show their understanding. A study conducted in a Scandinavian breast cancer online support group comprising 15 women showed that by sharing their personal stories with others in the group, participants were actively portraying their life stories and identities [9]. Similarly, a study conducted in another online support group for breast cancer patients examined the level of expression of negative emotions, showing that the possibility of conveying one’s negative feelings was imperative to ensure improvements in participants’ well-being and quality of life [10].

However, the effectiveness of online support group outcomes (eg, improvements in people’s mental health) remains largely unknown. One systematic review aimed to examine the effectiveness of online peer-to-peer support for young people with mental health problems; it identified six relevant studies to be reviewed: 3 randomized controlled trials, 2 pre-post studies, and one randomized trial [11]. These targeted studies examined various mental health–related issues, including depression and anxiety, general psychological problems, eating disorders, and substance use (ie, tobacco). Overall, two of the four randomized controlled trials yielded a positive effect for the peer-support group relative to the comparison group at postintervention: one targeting anxiety and the other targeting tobacco. The other two studies showed no evidence that a peer-to-peer support group was effective for eating disorders or depressive symptoms. Moreover, internet support groups are not risk-free; some of the risks are (1) hurtful comments could be posted, which could worsen the depression symptoms of those who read them or even encourage negative behaviors (eg, suicide attempts); (2) people could post inaccurate information; and (3) anecdotal personal stories about treatment may discourage or delay others from seeking treatment [12].

Importantly, online support groups do not always aim at effectiveness regarding distress relief outcomes [1]. Rather, they aim to promote emotional relief and an elevated sense of control. First, online support groups may challenge stigma. For example, online communities may serve as powerful venues where individuals with mental illness can challenge stigma through personal empowerment and increased hope [13]. Moreover, young adults with mental illness report that one of the primary reasons for connecting with others via the web is to feel less alone [14], because popular social media allows them to feel connected while providing a sense of relief from knowing that others share similar experiences and challenges [5]. Second, online support groups may increase consumer activation, which refers to the degree to which the individual understands that they must play an active role in managing their own health and health care, and the extent to which they feel able to fulfill that role [15]. For instance, learning from peers through web-based networks may help individuals with mental illness realize that they can make their own health care decisions [1]. In addition, previous studies have shown that when someone learns about other people’s personal experiences about facing an illness, they feel more confident in and empowered to make their own health care decisions [16]. In light of these delineations, we aimed to test these perspectives on the functions of online support groups.

### Study Setting

Owing to the rapid development of social networking services, online support groups take on different forms [1]. In particular, U2plus [17] is an online support group in which 23,864 users were registered (as of November 2019). It was developed to provide individuals with depression with structured peer-to-peer support while engaging in simple CBT exercises. Moreover, the name of the group has a phonetic trick to it, in that the pronunciation of *U2* in English is close to that of the Japanese word *Utsu*, which means *depression*, and the term *plus* is meant to suggest that *someone else is there for you*.

**Figure 1:**
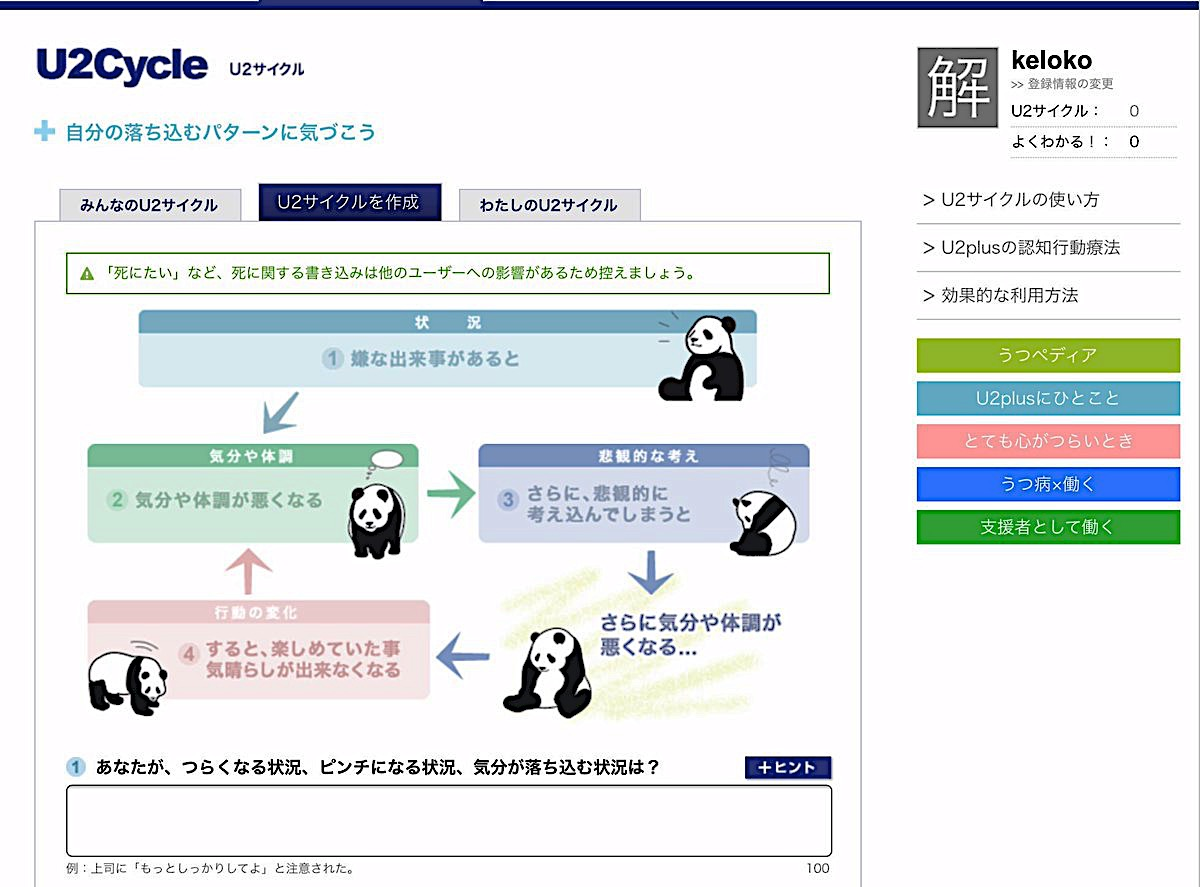
Screenshot of the U2Cycle function.

**Figure 2:**
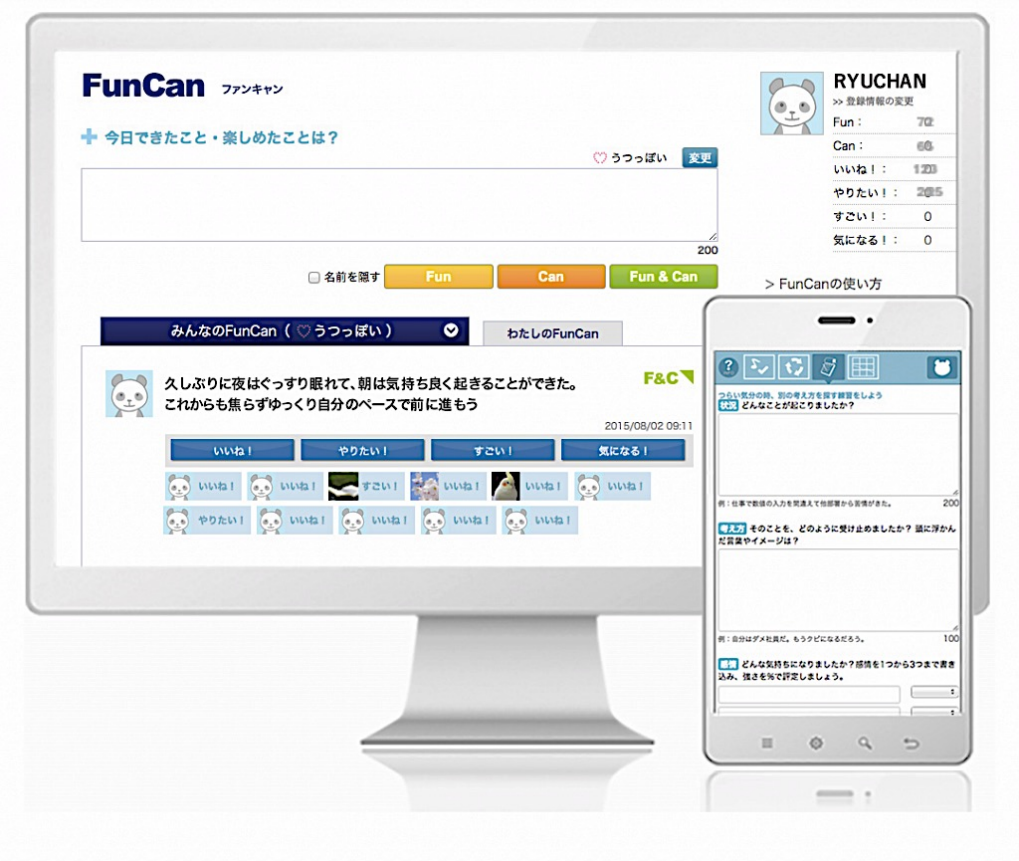
Screenshot of the FunCan function.

After signing up, the users gain access to the psychoeducation page, from which they can learn what depression is and what keeps depression going. By applying the information that they have learned from this page, users should be able to develop a simple formulation based on a cognitive model for depression on the U2Cycle page (Figure 1). On the F2Friend page, users can present their username, sex, age group, subjective level of recovery, a brief story of their depression (eg, how they developed depression, how they have been affected, and what they want to do when they recover), and messages to other users. These messages can be viewed by other users, and they can also respond to the messages by clicking the Like button. This resource was included because previous studies suggest that reading other people’s stories leads people to feel better informed and less anxious [18,19]. Furthermore, users can engage in simple CBT techniques. On the FunCan page (Figure 2), users can post whatever they have achieved and enjoyed in their everyday life [20], to which other users can respond by clicking the Like, Great, Interesting, and Want-to-do buttons when they view this activity. On the Column page, users can post a 5-column thought record, to which other users can respond to by clicking the Empathy button when they view it.

### Study Aim

This study aims to examine whether the use frequency of these U2plus functions is associated with decreased stigma, increased consumer activation, and depression levels.

## Methods

### Overview of the Survey

This section reports the nature of this web-based survey in accordance with the Checklist for Reporting Results of Internet E-Surveys [21]. The study was approved by the ethics committee of the International University of Health and Welfare (reference number 19-lg-46). The web-based survey was developed using Google Forms and was tested before fielding the questionnaire. In November 2019, 23,864 users registered in U2plus [17] were contacted by email and asked to voluntarily participate in a web-based survey by the end of that month (ie, before the COVID-19 outbreak). Participants assessed the survey and read the study information sheet before taking part. The survey had three screens, including the study information page, and the participants were able to review and change their answers through the back button. No incentives were offered to participants. No adaptations were made to the questionnaire, and the items were not randomized. Participants’ email addresses were collected to allow for duplicate entries from the same user to be found, but their IP addresses were not checked.

### Participants

In total, we had 350 participants (350/23,864, response rate 1.46%) willing to participate, including 219 women, 118 men, and 13 who did not disclose their gender, with a mean age of 38.36 (SD 10.095) years. Regarding the therapy they received, 88.0% (308/350) had been on medication for mental health problems and 66.6% (233/350) had received psychotherapy or mental health counseling.

### Measures

#### Overview

In addition to the standardized measures mentioned later, participants were asked about how long they had been using U2plus, to which they responded by choosing one of the five options: (1) less than 1 month, (2) 1 month to 6 months, (3) 6 months to 1 year, (4) 1 year to 3 years, and (5) over 3 years. Then they were asked how often they used each function of U2plus (ie, signing in, reacting to other users’ activities, reading U2Friend [other users’ profile and story], U2Cycle, FunCan, and Column), to which they responded by choosing 1 of 5 options: (1) never, (2) once a year, (3) once a month, (4) once a week, (5) a few times a week, and (6) almost every day.

#### Patient Health Questionnaire-9

Patient Health Questionnaire-9 (PHQ-9) [22] was used to measure participants’ general well-being. We used the Japanese version [23]. It is a 9-item self-reported questionnaire that was originally developed to measure depressive symptoms. Items are scored on a 4-point scale (0=not at all to 3=nearly every day), with total scores ranging from 0 to 27. The questions are based on the *Diagnostic and Statistical Manual of Mental Disorders, Fourth Edition* criteria, so it can provide both a diagnosis of depression and a measurement of its severity [23]. The internal consistency of the scale ranged from 0.86 to 0.89 in the original study [22].

#### Discrimination-Devaluation Scale

The Discrimination-Devaluation (D-D) scale [24] was used to measure participants’ public stigma. We used the Japanese version [25]. It asks people how much they agree with 12 statements that begin with “Most people believe [...],” “Most people think [...],” or “Most people would [...],” which are then followed by a relevant stereotype, an example of discrimination, or the opposite (ie, an accepting view or behavior). For this study, we modified the original D-D scale; in its original form, it refers to a “patient with mental health problems,” a “former patient with mental health problems,” or a person “who has been hospitalized for mental illness.” However, our adapted version referred to “a person who has received mental health treatment,” mainly because our objective was to measure perceived stigma toward a broader concept of mental health treatment, rather than stigma toward institutionalized treatment for severe mental illness. Items are scored on a 6-point Likert-type scale (0=strongly disagree to 5=strongly agree). As in the original, we constructed an index of perceived stigma by coding each response as 0, 1, 2, 3, 4, or 5, with higher numbers indicating higher perceived stigma. We then calculated the total score of the 12 items for each individual.

#### General Help-Seeking Questionnaire

The General Help-Seeking Questionnaire (GHSQ) [26] was used to measure participants’ future help-seeking intentions. The construct was measured by listing a number of potential help sources (ie, often used by people to help them deal with emotional and personal problems) and asking participants to indicate the likelihood of them seeking help from each source. Items are scored on a 7-point scale (1=extremely unlikely to seek help to 7=extremely likely to seek help). We then calculated their averages and divided them into two classifications: *Formal Sources*, computed by averaging the ratings for physicians, mental health professionals, and online counseling services, and *Informal Sources*, computed by averaging the ratings for partner, friends, and family.

### Data Analysis

We first calculated the frequency distributions of how long the participants had used U2plus and how often they used each U2plus function. After computing the means and SDs of PHQ-9, D-D scale, help-seeking intentions from formal sources, and help-seeking intentions from informal sources, we performed bilateral correlation analyses between these scales and the use frequency of U2plus functions. Subsequently, multiple regression analyses were conducted to test if the frequency use of each U2plus function uniquely predicted the D-D scale and GHSQ*.* All statistical analyses were performed using IBM SPSS Statistics 24.0. Two-sided analysis was adopted, and *P*<.05 was considered as statistically significant.

## Results

### Use Frequency for the U2plus Platform and for Each Function

The ratios of use frequency for the U2plus platform were: 10.3% (36/350) for less than 1 month, 10.9% (38/350) for 1 month to 6 months, 16.9% (59/350) for 6 months to 1 year, 33.4% (117/350) for 1 year to 3 years, and 28.6% (100/350) for over 3 years.

Table 1 summarizes the frequencies with which they used each function in the platform. The results suggested that 9.1% (31/341) of the participants signed in to U2plus almost every day, 5.9% (20/341) signed in more than once a week, and 6.7% (23/341) signed in once a week. Thus, approximately 21.7% (74/341) of the participants used U2plus once a week or more. On the other hand, almost half of the sample used U2plus only once a year. Similar trends were observed in some U2plus functions such as Reaction and FunCan, but participants used other functions less frequently, such as U2Cycle and Column.

**Table 1:** Participants’ use frequency for each of the U2plus functions.

Use frequency	Participant, n (%)					
	Never	Once a year	Once a month	Once a week	A few times a week	Almost every day
Signing in (n=341)	26 (7.6)	146 (42.8)	95 (27.9)	23 (6.7)	20 (5.9)	31 (9.1)
Reaction^a^ (n=334)	134 (40.1)	62 (18.6)	69 (20.7)	23 (6.9)	21 (6.3)	25 (7.5)
U2Friend^b^ (n=334)	118 (35.3)	99 (29.6)	72 (21.6)	15 (4.5)	21 (6.3)	9 (2.7)
U2Cycle^c^ (n=333)	177 (53.2)	83 (24.9)	59 (17.7)	11 (3.3)	2 (0.6)	1 (0.3)
FunCan^d^ (n=335)	130 (38.8)	67 (20)	72 (21.5)	23 (6.9)	20 (6)	23 (6.9)
Column^e^ (n=336)	177 (53.2)	83 (24.9)	59 (17.7)	11 (3.3)	2 (0.6)	1 (0.3)

^a^Reacting to other users’ activities.

^b^Reading other users’ profiles and stories.

^c^A cognitive behavioral therapy exercise through which users can develop a simple formulation.

^d^A cognitive behavioral therapy exercise through which users can post what they had achieved or enjoyed.

^e^A cognitive behavioral therapy exercise through which participants can write a 5-column thought record.

### Relationships Between U2plus Function Use Frequencies, Depression, Stigma, and Help-Seeking Behavior

Table 2 presents the means and SDs of participants’ scores on each scale and their correlations with participants’ use frequency for each function of the U2plus platform. Regarding the relationships with the different scales, PHQ-9 was weakly and positively correlated with the use of the U2Cycle (*P*=.02) and Column (*P*=.003) functions. The *Formal Sources* variable of the GHSQ was weakly correlated with the use of signing in (*P*=.04) and FunCan (*P*=.04) functions. Finally, Table 3 presents the results of multiple regression analyses, which revealed that no use of the U2plus function uniquely predicted the D-D scale or GHSQ.

**Table 2:** Mean (SD) and correlations to the frequency of participants’ activity.

Values and U2plus functions	Values, mean (SD)	Correlation factor					
		Signing in	Reaction^a^	U2Friend^b^	FunCan^c^	Column^d^	U2Cycle^e^
PHQ-9^f^	12.38 (6.95)	0.048	0.072	0.099	0.046	0.161^g^	0.125^h^
D-D^i^ scale	48.97 (10.42)	0.001	–0.015	–0.018	–0.005	0.027	0.042
GHSQ^j^-formal^k^	4.53 (1.27)	0.110^l^	0.103	0.063	0.111^l^	0.037	0.049
GHSQ-informal^m^	3.72 (1.33)	–0.032	–0.036	–0.011	–0.02	–0.045	–0.048

^a^Reacting to other users’ activities.

^b^Reading other users’ profiles and stories.

^c^A cognitive behavioral therapy exercise through which users can post what they had achieved or enjoyed.

^d^A cognitive behavioral therapy exercise through which participants can write a 5-column thought record.

^e^A cognitive behavioral therapy exercise through which users can develop a simple formulation.

^f^PHQ-9: Patient Health Questionnaire-9.

^g^*P*=.003.

^h^*P*=.02.

^i^D-D: Discrimination-Devaluation.

^j^GHSQ: General Help-Seeking Questionnaire.

^k^Help-seeking intentions from formal sources.

^l^*P*=.04.

^m^Help-seeking intentions from informal sources.

**Table 3:** Multiple regression analyses of stigma and help-seeking predicted by frequency use of the U2plus functions.

Dependent variable	D-D scale^a^				GHSQ^b^-formal^c^				GHSQ-informal^d^		
	B	β	*t* test (*df*)	B		β	*t* test (*df*)	B		β	*t* test (*df*)
Signing in	0.015	.002	0.015 (321)	0.084		.091	0.671 (321)	–0.073		–.076	–0.556 (321)
Reaction^e^	0.617	.092	0.556 (321)	0.072		.087	0.530 (321)	0.141		.163	0.987 (321)
U2Friend^f^	0.135	.014	0.126 (321)	–0.108		–.089	–0.827 (321)	–0.031		–.024	0.223 (321)
FunCan^g^	0.787	.072	0.721 (321)	0.038		.028	0.285 (321)	–0.054		–.038	–0.384 (321)
Column^h^	–0.783	–.119	–0.674 (321)	0.013		.016	0.089 (321)	–0.127		–.150	–0.848 (321)
U2Cycle^i^	–0.313	–.04	–0.391 (321)	–0.046		–.048	–0.472 (321)	0.087		.086	0.849 (321)

^a^D-D: Discrimination-Devaluation.

^b^GHSQ: General Help-Seeking Questionnaire.

^c^Help-seeking intentions from formal sources.

^d^Help-seeking intentions from informal sources.

^e^Reacting to other users’ activities.

^f^Reading other users’ profiles and stories.

^g^A cognitive behavioral therapy exercise through which users can post what they had achieved or enjoyed.

^h^A cognitive behavioral therapy exercise through which participants could write a 5-column thought record.

^i^A cognitive behavioral therapy exercise through which users can develop a simple formulation.

## Discussion

### Principal Findings

Regarding the relationships between U2plus function use frequency, stigma, and help-seeking behavior, our results highlighted mixed outcomes: the D-D scale and help-seeking intention from informal sources were not associated with any use frequency of U2plus functions. The use frequency of the signing in and FunCan functions was correlated only with help-seeking intentions from formal sources. However, these use frequencies did not predict help-seeking intentions from formal sources in the multiple regression analysis.

We also analyzed participants’ demographic characteristics to better understand the personal characteristics of the average user of the U2plus platform, namely, those who use online support groups for depression. Participants’ average score in PHQ-9 was 12.38, suggesting that those who use the platform may be moderately depressed. Moreover, almost 88% (308/350) of the participants had been medicated for their mental health problems, suggesting that most users may be looking for something other than (or in addition to) pharmacological interventions to help them deal with their mental health issues. About 21.7% (74/341) of the participants signed in and used FunCan and Reaction more than once a week. This suggests that some users use U2plus on a regular basis, and they use simpler functions such as posting whatever they have achieved and enjoyed over the past few days, and responding to those posts, than other functions such as U2Cycle and Column, which may require more time and effort. In addition, depression measured by PHQ-9 was positively associated with the frequency use of Column and U2Cycle. Thus, for other users, the U2plus platform may be more of an *on-demand* service (ie, they use it whenever they need or feel like it).

### Comparison With Prior Work

Our results showed that stigma was not associated with any of the functions of the U2plus platform. This suggests that U2plus users may feel normalized when they discover that many people have depression, as well as when they share their experiences; however, they are constrained by the limitations imposed by the platform; for example, they can only react to other users’ posts by clicking the *Like* button, to prevent users from writing negative comments to each other. This is in line with two studies: one showing that loneliness and social support did not change by using internet support groups for depression [27] and another showing that, among participants who used internet depression support groups, social support scores did not change during follow-up [28], although neither of these studies directly measured stigma, but loneliness and social support, which may affect stigma. However, they found that seeking emotional support was the most popular reason for using an internet support group [28].

Our results also showed that the use frequency of the signing in and FunCan functions of the U2plus platform were associated with (but did not uniquely predict) help-seeking intentions from formal sources. These results may not fully support a notion proposed by previous study, that is, when going to a medical visit, having to undergo hospitalization, or learning about others’ experiences may help individuals to feel more at ease with the situation and have a better understanding of what questions need to be asked and what to expect [1]. Many people tend to seek health information or alternative treatment options on web-based platforms after feeling dissatisfied or in clear disagreement with the advice put forth by a physician during a medical visit [29]. Confirming this statement and highlighting the positive aspects of online support groups, a study showed that peer-facilitated approaches may be important strategies for improving the quality of health care encounters, as these approaches help patients to find better ways to communicate with physicians, better navigate in unfamiliar health care environments, and take an active role during primary care visits [30].

### Limitations and Future Directions

First, we asked all users of U2plus for their participation; this means that our participants might have had various mental health problems, not only depression. Owing to the low response rate, the sample may not have been representative of the users registered to the program in terms of basic demographic information. Thus, future studies need to recruit individuals with a specific mental health problem and establish their diagnosis by conducting structured clinical interviews. Second, we relied on participants’ self-reports about how often they used each U2plus function; thus, future studies are warranted to measure these frequencies in more objective ways, as we were not allowed to track users’ operation histories. Third, we used a cross-sectional design; thus, future studies need to use a longitudinal design and examine how these variables fluctuate with time as participants use these online support groups.

Fourth, we tested the effects of an online support group on only stigma and help-seeking intentions; therefore, future studies should examine other variables, such as sense of control, self-confidence, sense of independence, and social interaction [2]. Finally, participants were limited to those who had a good understanding of the Japanese language, and the U2plus platform was not what most would call a *typical* online support group regarding user interactions, as users were able to engage in some simple CBT exercises. Yet, although this is a limitation, it also raises research questions regarding what kind of online support group would be suitable for specific types of mental health problems.

### Conclusions

Our findings revealed that most of the U2plus users were receiving pharmacological treatment and used U2plus as an alternative treatment option, with approximately 21.7% (74/341) of them signed in to it on a regular basis. In addition, the use frequency of some functions was correlated with help-seeking intentions from formal sources. Future studies need to more closely investigate how online support groups can help mental health stakeholders, including those who are on medication but unable to fully recover, those who are on the waiting list for evidence-based psychological treatment, and those who try to prevent relapse after recovery.

## Abbreviations

CBT: cognitive behavioral therapy
D-D: Discrimination-Devaluation
GHSQ: General Help-Seeking Questionnaire
PHQ-9: Patient Health Questionnaire-9

## References

[1] Andersson G (2014). The Internet and CBT: A Clinical Guide.

[2] Barak A, Klein B, Proudfoot JG (2009). Defining internet-supported therapeutic interventions. Ann Behav Med.

[3] Gowen LK (2013). Online mental health information seeking in young adults with mental health challenges. J Technol Hum Serv.

[4] Spinzy Y, Nitzan U, Becker G, Bloch Y, Fennig S (2012). Does the internet offer social opportunities for individuals with schizophrenia? A cross-sectional pilot study. Psychiatry Res.

[5] Kummervold PE, Gammon D, Bergvik S, Johnsen JK, Hasvold T, Rosenvinge JH (2002). Social support in a wired world: use of online mental health forums in Norway. Nord J Psychiatry.

[6] Haker H, Lauber C, Rössler W (2005). Internet forums: a self-help approach for individuals with schizophrenia?. Acta Psychiatr Scand.

[7] Pfeiffer PN, Heisler M, Piette JD, Rogers MA, Valenstein M (2011). Efficacy of peer support interventions for depression: a meta-analysis. Gen Hosp Psychiatry.

[8] Esterling BA, L'Abate L, Murray EJ, Pennebaker JW (1999). Empirical foundations for writing in prevention and psychotherapy: mental and physical health outcomes. Clin Psychol Rev.

[9] Høybye MT, Johansen C, Tjørnhøj-Thomsen T (2005). Online interaction. Effects of storytelling in an internet breast cancer support group. Psychooncology.

[10] Lieberman MA, Goldstein BA (2006). Not all negative emotions are equal: the role of emotional expression in online support groups for women with breast cancer. Psychooncology.

[11] Ali K, Farrer L, Gulliver A, Griffiths KM (2015). Online peer-to-peer support for young people with mental health problems: a systematic review. JMIR Ment Health.

[12] Goodwin BC, Ford DE, Hsiung RC, Houston TK, Fogel J, Van Voorhees BW (2018). First, do no harm: referring primary care patients with depression to an internet support group. Telemed J E Health.

[13] Lawlor A, Kirakowski J (2014). Online support groups for mental health: a space for challenging self-stigma or a means of social avoidance?. Comput Hum Behav.

[14] Burns JM, Durkin LA, Nicholas J (2009). Mental health of young people in the United States: what role can the internet play in reducing stigma and promoting help seeking?. J Adolesc Health.

[15] Hibbard JH, Mahoney ER, Stockard J, Tusler M (2005). Development and testing of a short form of the patient activation measure. Health Serv Res.

[16] Entwistle VA, France EF, Wyke S, Jepson R, Hunt K, Ziebland S, Thompson A (2011). How information about other people's personal experiences can help with healthcare decision-making: a qualitative study. Patient Educ Couns.

[17] U2plus.

[18] Setoyama Y, Yamazaki Y, Namayama K (2011). Benefits of peer support in online Japanese breast cancer communities: differences between lurkers and posters. J Med Internet Res.

[19] van Uden-Kraan CF, Drossaert CH, Taal E, Seydel ER, van de Laar MA (2008). Self-reported differences in empowerment between lurkers and posters in online patient support groups. J Med Internet Res.

[20] Kingdon DG, Turkington D (2004). Cognitive Therapy of Schizophrenia.

[21] Eysenbach G (2004). Improving the quality of web surveys: the Checklist for Reporting Results of Internet E-Surveys (CHERRIES). J Med Internet Res.

[22] Kroenke K, Spitzer RL, Williams JB (2001). The PHQ-9: validity of a brief depression severity measure. J Gen Intern Med.

[23] Kroenke K, Spitzer RL (2002). The PHQ-9: a new depression diagnostic and severity measure. Psychiatr Ann.

[24] Link BG (1987). Understanding labeling effects in the area of mental disorders: an assessment of the effects of expectations of rejection. Am Sociol Rev.

[25] Shimotsu S, Sakamoto S, Horikawa N, Sakano Y (2006). Reliability and validity of the Japanese version of link's devaluation-discrimination scale. Jpn J Psychiatr Treat.

[26] Wilson CJ, Deane FP, Ciarrochi JV, Rickwood D (2005). Measuring help seeking intentions: properties of the general help seeking questionnaire. Can J Couns.

[27] Houston TK, Cooper LA, Ford DE (2002). Internet support groups for depression: a 1-year prospective cohort study. Am J Psychiatry.

[28] Raupach JC, Hiller JE (2002). Information and support for women following the primary treatment of breast cancer. Health Expect.

[29] Masumoto S, Sato M, Maeno T, Ichinohe Y, Maeno T (2017). Association between potentially inappropriate medications and anxiety in Japanese older patients. Geriatr Gerontol Int.

[30] Bartels SJ, Aschbrenner KA, Rolin SA, Hendrick DC, Naslund JA, Faber MJ (2013). Activating older adults with serious mental illness for collaborative primary care visits. Psychiatr Rehabil J.

